# Overlooked Scents: Chemical Profile of Soma, Volatile Emissions and Trails of the Green Tree Ant, *Oecophylla smaragdina*

**DOI:** 10.3390/molecules25092112

**Published:** 2020-04-30

**Authors:** Vivek Kempraj, Soo Jean Park, Stefano De Faveri, Phillip W. Taylor

**Affiliations:** 1 Applied BioSciences, Macquarie University, Sydney 2109, NSW, Australia; soojean.park@mq.edu.au (S.J.P.); Phil.Taylor@mq.edu.au (P.W.T.); 2 Horticulture and Forestry Science, Queensland Department of Agriculture and Fisheries, Mareeba 4880, QLD, Australia; stefano.defaveri@daf.qld.gov.au

**Keywords:** *Oecophylla smaragdina*, cuticular hydrocarbons, headspace volatiles, chemical ecology

## Abstract

The green tree ant, *Oecophylla smaragdina*, is one of only two recognized species of weaver ants. While the identity and functions of chemicals produced and emitted by its congener *O. longinoda* have been studied quite extensively and serve as a valuable model in chemical ecology research, little comparable information is available about *O. smaragdina*. Although some analyses of chemicals produced and emitted by *O. smaragdina* have been reported, the literature is fragmentary and incomplete for this species. To address this knowledge gap, and to enable comparisons in the chemical ecology of the two weaver ant species, we here describe diverse chemicals from the cuticle, Dufour’s glands, poison glands, head, headspace volatiles, and trails of *O. smaragdina*.

## 1. Introduction

The weaver ants comprise two recognized species: [1] *Oecophylla longinoda*, which is mainly found in the tropical regions of Africa and [2] *O. smaragdina* (green tree ant), which is found in the tropical regions of India, Southeast Asia, and Australia. *Oecophylla longinoda* has been the subject of extensive research over many decades, including significant focus on its chemical ecology [1]. This species’ socio-chemical system [2,3,4], glandular chemicals [5,6], trail pheromone [3], territorial pheromone [2], and alarm/communication system including volatiles in Dufour and poison glands [6,7,8] have all been well-documented. In contrast, while observations suggest a similar importance of olfaction-mediated behavior in *O. smaragdina*, very little is known about the identity or function of chemicals produced and released by this species [1,9,10]. The few existing studies of *O. smaragdina* chemical profile are restricted to Dufour’s glands, mandibular glands [11], head, and the gaster [12]. However, these studies are mostly based on small sample size (*n* = 2 to 6) [11] or relied on crude extracts [12]. Studies of cuticular chemistry have considered chemical mimicry of *O. smaragdina* by spiders, but the extraction method was reliable mainly for heavy cuticular hydrocarbons and was inefficient for lighter compounds that tend to be important for communication [13,14]. Major workers make up 90% of a *O. smaragdina* colony and are the communication line between the nest and the external environment. Here we present a comprehensive chemical profile of the major workers of *O. smaragdina*.

## 2. Results and Discussion

**Figure 1:**
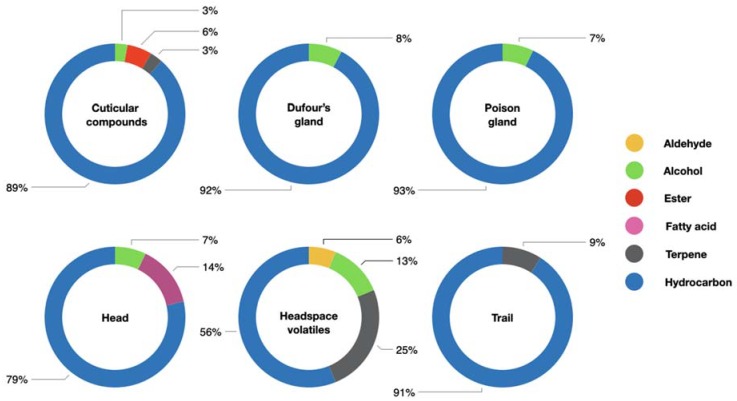
Composition of compounds isolated from the green tree ant, *Oecophylla smaragdina*. The dominant components were hydrocarbons, with other compounds being less abundant.

Previous studies of *O. smaragdina* chemical ecology have yielded a fragmentary and incomplete knowledge of the compounds produced and emitted by this ecologically and economically important ant species [15,16,17]. We identified a total of 59 compounds from the cuticle, Dufour’s gland, poison gland, head, headspace volatiles, and trails of *O. smaragdina* workers, including aldehydes, alcohols, carboxylic acid, esters, fatty acids, terpenes and hydrocarbons (Figure 1). This analysis provides essential foundations for functional studies investigating the role of specific compounds, as well as investigation of regional and colony variation in chemical profile.

### 2.1. Identification of Compounds from Cuticle of Oecophylla Smaragdina

Cuticular extracts contained several straight and methyl branched hydrocarbons (89%), esters (6%), terpene (3%), and alcohols (3%). An ant’s cuticle usually contains between 5 and 50 complex chemical compounds, but in extreme cases may contain hundreds of compounds [18]. In addition to having a wide range of cuticular compounds to protect against dehydration [18,19], ants also make extensive use of cuticular compounds for inter and intra-species communication [20,21,22,23]. The reliance on odor for nestmate recognition can make ants vulnerable to exploitation by enemies. For example, a salticid spider, *Cosmophasis bitaeniata*, has evolved an ability to mimic cuticular compounds of *O. smaragdina* in order to avoid detection by ants while foraging on ant larvae [14]. This study reported 14 straight and branched hydrocarbons from *O. smaragdina* cuticles but had overlooked lighter compounds that may be crucial in inter and intra-specific communication. We detected 31 hydrocarbons (Table 1) with undecane, heneicosane, and tricosane being the major compounds. We employed a shorter extraction time (10 s) compared to (10 min extraction + 1 min agitation) in order to extract lighter cuticular compounds that were overlooked previously [14]. The previous study mainly focused on heavier hydrocarbons C_29_ to C_37_ [14]. We also found two wax esters, namely hexyl formate and octyl formate (Table 1), that were not reported previously. Wax esters are major constituents of cuticular lipids in most insects [24]. Although they may serve a variety of biological functions, their major function is to reduce evaporative water loss [25,26,27]. A terpene, limonene was present at low concentrations (0.02%) in the cuticular extract. Keegans et al. has previously reported the presence of limonene in the Dufour and mandibular glands of *O. smaragdina* [11]. Ants allogroom nest mates to keep the colony free from disease [28], and these compounds were likely dispersed over the ant’s cuticle during allogrooming [28]. An alcohol, 1-Octanol, was also found in the cuticule of *O. smaragdina* and having been identified previously in the mandibular glands of *O. smaragdina*, likely also was transferred to the cuticle during allogrooming [11].

### 2.2. Identification of Compounds from Dufour’s Gland and Poison Gland

Dufour’s glands and poison glands contained a mixture of hydrocarbons (92% and 93%, respectively) and alcohols (8% and 7%, respectively). However, the concentration of individual compounds varied. Undecane (27.45%), heneicosane (16.07%), and tricosane (25.83%) were the dominant hydrocarbons and 1-Heptadecanol was the only alcohol present in the Dufour glands (Table 2 and Table 3). However, poison glands contained undecane (52.68%) as the largest component followed by tricosane (15.65%) and heneicosane (5.89%). Previous work on secretions from Dufour’s gland in *O. smaragdina* [11] reported undecane (41.4%) and heneicosane (13.8%) as the major compounds, whereas tricosane was reported in small amounts. While there are no previous studies of compounds from poison glands of *O. smaragdina*, studies of *O. longinoda* reported undecane (25–28%) to be the major compound [7]. While functions have not been investigated in *O. smaragdina*, Dufour’s and poison glands are central to the alarm system of *O. longinoda*. Chemicals secreted by these glands are used in combination for communication, territory and trail marking, and alarm signals [6]. For example, the alarm volatiles of *O. longinoda* consists of a blend n-undecane from Dufour’s glands and formic acid from the poison gland. The main components from the two glands act synergistically to elicit an alarm reaction in *O. longinoda* [6,7].

### 2.3. Identification of Compounds from Head

Compounds extracted from the head constituted of straight and methyl branched hydrocarbons (76%), alcohols (7%), and fatty acids (17%). Hydrocarbons in the head were mostly similar to hydrocarbons in cuticular extracts and may be mostly from the cuticle of the head. The head also contained two alcohols, 1-hexanol and 1-octanol, which were previously identified in the mandibular glands and head extract of *O. smaragdina* [11,12] and *O. longinoda* [6]. Although alcohols have been identified in other ant species (*Crematogaster* sp.), the biological functions remain unknown [36,37,38]. Fatty acids were present only in the head extract in our study (Table 4). The identified fatty acids in head extracts, including palmitic acid, linoleic acid, oleic acid, and stearic acid are ubiquitous in nature and are known to have antifungal properties [39]. In the ant *Solenopsis invicta*, such fatty acids can increase in amount on the body surface after death and provide a ‘death cue’ that induces necrophoric behavior [40,41,42,43].

### 2.4. Identification of Compounds from Headspace

Headspace volatiles from *O. smaragdina* included hydrocarbons (56%), aldehyde (6%), alcohol (13%) and terpenes (25%) and was the only extract that contained diverse terpenes and terpinoids such as *p*-cymene, limonene, γ-terpinene, and dihydromyrcenol (Table 5). Terpenes of *O. smaragdina* have been previously reported from extracts of mandibular glands [11]. It was previously thought that terpenes including limonene, *p*-cymene and γ-terpinene may be sequestered by ants [50] for use in alarm signals [51]. However, recent studies have shown that terpenes are produced by the ants themselves [52]. Headspace volatiles have not previously been investigated in *Oecophylla* species and so species comparisons are not possible at this time. Given the importance of headspace volatiles as mediators of interactions, comparative data from *O. longinoda* would be useful.

### 2.5. Identification of Compounds from Trail

**Figure 2:**
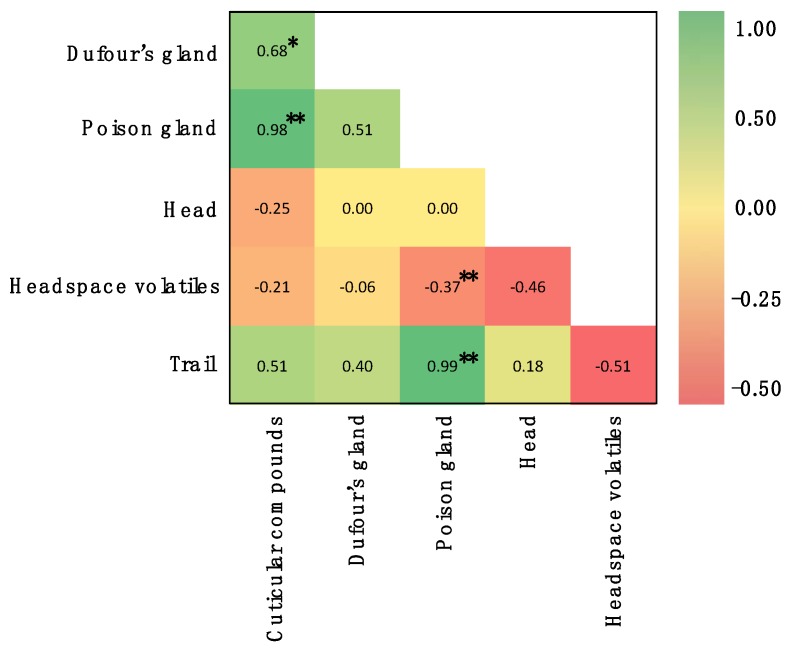
Multivariant correlation analysis of compounds isolated from different body parts, headspace, and trails of *Oecophylla smaragdina*. Analysis was based on the concentration and presence/absence of compounds. Pearson *R* value and *P* value were used as benchmark to determine the origin of compounds. Significance is denoted by * (<0.01); ** (<0.001).

Trails consist of chemicals laid by worker ants from food site and back to the nest. Many studies have demonstrated the presence of trail chemicals in *O. smaragdina* and *O. longinoda* [2,4,57] but the identity of these chemicals has not been investigated previously in either species. Therefore, in this study, we analyzed trail of *O. smaragdina* from trail washes of major workers. Trail washes contained hydrocarbons (91%) with undecane, nonacosane and hentriacontane being the major compounds (Table 6) and terpenes (9%; limonene) which the ants may use along with hydrocarbons for recruitment [4]. Next, to ascertain the likely source of the trail compounds we conducted a multivariant correlation analysis of chemicals from different sources of the ant (Figure 2). The results suggest that Dufour’s and poison glands are the likely source of trail compounds [58]. This analysis also found that chemicals extracted from the ant’s head and headspace volatiles tended to be unique, and further studies are needed to ascertain their origins.

## 3. Materials and Methods

### 3.1. Chemicals and Standards

Authentic standards of 1-hexanol, formic acid hexyl ester, hexanoic acid, *p*-cymene, limonene, γ-terpinene, 1-octanol, dihydromyrcenol, 1-undecene, nonanal, formic acid octyl ester, palmitic acid, linoleic acid, oleic acid, stearic acid, alkane C_8_–C_40_ standard and hexane were purchased from Sigma-Aldrich, Alfa-Aesar, or TCI. All chemicals were of analytical grade (≥98% purity) and were used without further purification.

### 3.2. Insects

Green tree ants (major workers) were collected in August 2018 from 5 different colonies at the Mareeba Research Facility, Queensland Department of Agriculture and Fisheries, QLD, Australia (17.00724 °S, 145.42984 °E). Colonies were differentiated based on distance (~300 m) between colonies, canopy interconnections and interconnection trails [59]. Insects were sampled from different nests within a colony and were directly extracted or dissected in the laboratory of the Mareeba Research Facility. The collected samples were transported to Macquarie University, Sydney and prepared for GCMS analysis. The collected samples were stored at stored at −20 °C until transported via a local courier service. All the preparation and analysis of GCMS samples were conducted at Macquarie University. The samples were immediately processed as received that involved removing aqueous droplet(s), filtering off solid matters and evaporating solvents under a nitrogen stream to a small volume and stored at 4 °C until GCMS analysis.

### 3.3. Cuticular Compounds

Cuticular compounds (CCs) of social insects commonly function in communication of colony identity and in maintaining social unity. In ants, CCs are spread throughout the epi-cuticular surface are commonly essential for nestmate recognition [60]. Heavier CCs from green tree ants were previously reported by Allan et al. (2002), but our focus was to analyze lighter CCs that were previously overlooked. The chromatogram reported by Allen et al. showed many peaks at early retention times, but these were not reported [14]. Therefore, we used a shorter extraction time than that of Allan et al. in order to focus on lighter CCs [14]. Ant samples comprised individuals (*n* = 100) dipped in 10 mL of hexane for 10 s. A total of 27 samples were collected.

### 3.4. Dufour’s and Poison Glands

**Figure 3:**
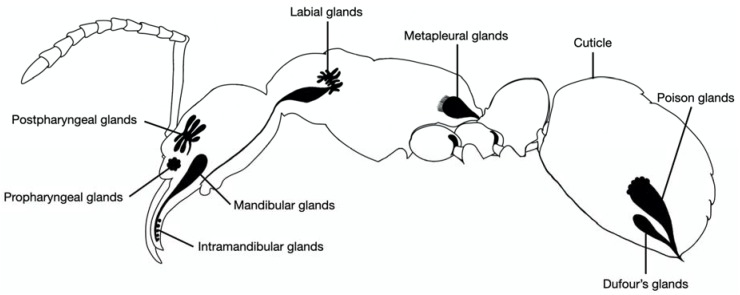
Schematic diagram of green tree ant’s exocrine glands.

Ants were collected in 50 mL plastic vials and placed in a freezer (−20 °C) for 10 min to kill them. Dufour’s glands were extracted by dissecting the last segment of abdomen. The remnant tissues around the gland were carefully removed using fine forceps. The clean glands were immediately placed into 1.5 mL of hexane and crushed. Each sample contained ten glands. The poison gland is located in the abdomen, beside the Dufour’s gland (Figure 3), hence dissection and collection were the same as for the Dufour’s gland. A total of 10 samples of the two glands were collected.

### 3.5. Head

Heads of ants contain many glands and are rich in volatile compounds. Many compounds from the head are known to be used for communication or defense [61]. Ant heads contain complex exocrine structures associated with mouthparts and the antennae. They also contain many glands, but four glands are particularly large, namely the mandibular gland, intramandibular glands, the propharyngeal gland, and the postpharyngeal gland (Figure 3) [61]. As it is difficult to dissect and separate individual glands from the head, we used the whole head in this study. Collected green ants were killed by placing them in a −20 °C freezer. Heads were removed with dissection scissors and immediately placed in 1.5 mL of hexane in a glass vial. This was repeated until ten heads were collected per sample. A total of seven samples were collected.

### 3.6. Headspace Volatiles

Volatile compounds present in the air surrounding an insect can be important for mediation of intra- and inter-specific interactions. An air entrainment system with the capacity to sample 10 chambers was used to collect headspace volatiles of live ants. A cylindrical glass chamber (150 mm long × 40 mm ID) with an inlet and outlet at the ends was used to contain ants. A charcoal filter was connected to the inlet (4 mm ID) of the glass chamber using Tygon tubing (E-3603). The outlet of the glass chamber was connected to a 6 × 50 mm glass tube containing an adsorbent (50 mg, Scientific Instrument Services Inc, Tenax-GR Mesh 60/80) fitted to a screw cap with O-ring. Ten ants were placed inside the glass chamber and were allowed to acclimatize for 30 min prior to collection of volatiles. Nine chambers containing ants and one empty control chamber were set up for each run. Headspace volatiles were adsorbed into Tenax at a flow rate of 0.5 L/min for 30 min. Green tree ants were highly active during afternoons; therefore, all collections were conducted between 2 and 4 pm. The adsorbed volatiles were eluted with 1 mL of hexane into a clean 1.5 mL sample vial. A total of 36 samples were collected. A negative control in each experiment was used to identify any background impurities.

### 3.7. Trails

Trail chemicals are important for co-ordination of foraging in many ants [62]. *Oecophylla smaragdina* inhabit hot and humid tropical ecosystems and forage over long distances across the tree canopy and the ground [63]. Given the required persistence of trails in wet conditions, their trail chemicals are expected to be of quite low volatility and to have low solubility in polar solvents such as water. Green tree ants at Mareeba Research Facility had nests close to a metal mesh fence. This mesh fence served as a regular path to transport food and other materials to the nest. Prior to collection, the section of fence (~3 m) that the ants used to commute was rinsed with acetone (100 mL) to remove pre-existing trail chemicals and contaminants. The ants were allowed to make a trail on the cleaned section of the mesh for 24 h. Next, during periods of high ant activity, the metal wire was rinsed, section by section, with a total of 100 mL hexane into a beaker (500 mL). A same length of fence not used by the ants was used as a control. The trail and control washes were concentrated under a gentle stream of clean air from a compressor with filters to remove water and oil (Pilotair K12, Pilotair, NSW, Australia) down to approximately 10 mL. A total of ten samples were collected.

### 3.8. Sample Processing

CCs, Dufour’s glands, poison glands and head samples contained minute quantities of water/debris and these were removed by adding a drying agent (sodium sulfate) and by gravity filtration of the collected solutions. Samples free from water and debris were concentrated under a gentle stream of nitrogen gas. CC samples were concentrated to 1 mL while Dufour’s glands, poison glands and head samples were concentrated to 0.5 mL. Trail samples were filtered to remove solid matter and concentrated to 1 mL under a gentle stream of nitrogen gas. Headspace volatile samples did not require further processing.

### 3.9. Gas Chromatography–Mass Spectrometry (GC–MS) Analysis

GC–MS analysis was carried out on a Shimadzu GC–MS TQ8030 spectrometer (Shimadzu Corporation, Kyoto, Japan) equipped with a split/splitless injector and SH RTX-5MS (30 m × 0.25 mm, 0.25 µm film) fused silica capillary column. The carrier gas was helium (99.99%) at a flow rate of 1 mL/min. An aliquot of 1 µL sample was injected in splitless mode with injector temperature of 270 °C. The temperature program for CCs, head extracts and trail samples were as follows: 50 °C for 1 min to 280 °C at 10 °C·min^−1^, then increased to 300 °C at 2 °C·min^−1^. The temperature program for Dufour’s gland, poison gland, and headspace extracts were as follows: 50 °C for 1 min to 280 °C at 10 °C·min^−1^, then increased to 300 °C at 5 °C·min^−1^. The ion source and transfer line temperatures were 200 °C and 290 °C respectively. The ionization method was electron impact at a voltage of 70 eV. The spectra were obtained over a mass range of *m*/*z* 45–650. Hexane solvent runs obtained from each batch of an average 20 runs were used as controls to eliminate impurities from the analyses. The instrument maintenance and alkane calibration runs were routinely performed to prevent any technical issues. The relative amounts of compounds were calculated by dividing the peak area of a compound by the sum of peak area of all compounds. The data were presented as average percentages of replicates.

For identification, the mass spectra were analyzed by Shimadzu GC–MS post-run and compared with NIST library (NIST17-1, NIST17-2, NIST17s) to suggest candidates of the compounds. The purchased compounds were co-analyzed to confirm assignment. Mass fragmentation ions were analyzed in conjunction with comparing experimental Kovat indices and that published in the literature to assign compounds. For the structural assignments of methyl branched hydrocarbons, the chain length and the number of inner branched methyl groups were established by examining an equivalent chain length and molar mass of a compound. Molecular ions of inner branched hydrocarbons often do not appear or are weak in mass spectra and hence a molar mass of a branched hydrocarbon was established by examining fragmentations of M − 15, M − 29 and so on. The fragmentation of a branched hydrocarbon generates not only odd mass, but also mass secondary ions by hydride transfer if the chain length is sufficiently long. Intensities of these ions depend on whether a secondary fragment ion has an inner branch and on the carbon chain length of such an ion. Hence, these generalizations of mass peaks of branched hydrocarbons described by previous studies were used to assign the identity and branch positions of a hydrocarbon [64,65,66].

### 3.10. Data Analysis

The mean of relative abundance of compounds detected from each body part, headspace volatile and trail were subjected to multivariant correlation analysis using Microsoft Excel for Mac (version 16.3, Microsoft Corporation, Redmond, WA, USA). A correlation matrix was constructed to understand the source of the compounds. The matrix contains Pearson R value.

## 4. Conclusions

The present study is the first to have systematically identified chemicals from different body parts and glands of *O. smaragdina.* This study provides a foundation for more detailed investigation of the function of compounds produced and emitted by *O. smaragdina*, as well as investigation of regional and colony variation. In addition to broad comparisons between the two recognized species of *Oecophylla*, there is ongoing debate about the status of regional variations. For example, *O. smaragdina* is red in India and green in South-East Asian countries and Australia. Some authors advocate division of *O. longinoda* into eight subspecies and *O. smaragdina* into six subspecies [1]. Regardless of the taxonomic status of such regional variation, it is reasonable to anticipate geographic variation in chemical profiles both at a geographic scale such as might be consistent with regional/subspecies variation, or at a smaller scale as colony variation. Such variation may reflect heritable differences, but may also reflect differences in environmental influences, such as from nutrition [67]. In *O. longinoda*, the reported chemical profiles exhibit considerable variation based both on geography and between colonies [6,8], and differences between colonies have also been reported in *O. smaragdina* [59]. The present study provides a strong foundation for future investigations of regional and colony variation, and functional chemical ecology, of *O. smaragdina*.

## Figures and Tables

**Table 1:** Cuticular compounds (CCs) isolated from workers of *Oecophylla smaragdina*.

Compounds	MW	RT (min)	RI	Lit. RI [Ref]	ECL	Content (%)
						(Mean ± SE)
Formic acid hexyl ester	130.18	5.02	929	927 [29]	9.29	1.41 ± 0.29
Decane	142.28	6.15	1004		11.04	0.69 ± 0.10
Limonene	136.23	6.63	1035	1044 [30]	10.35	0.02 ± 0.001
1-Octanol	130.23	7.22	1073	1068 [31]	10.73	0.05 ± 0.01
1-Undecene	154.29	7.53	1093	1090 [32]	10.93	0.10 ± 0.02
Undecane	156.31	7.67	1101		11.01	38.12 ± 3.72
Formic acid, octyl ester	158.24	8.12	1131	1110 [33]	11.31	0.16 ± 0.04
Dodecane	170.33	9.17	1201		12.01	0.68 ± 0.11
1-Tridecene	182.35	10.39	1288	1288 [32]	12.88	0.51 ± 0.08
Tridecane	184.36	10.58	1301		13.01	5.65 ± 0.83
Tetradecane	198.39	11.92	1401		14.01	0.11 ± 0.02
Pentadecane	212.41	13.19	1501		15.01	4.18 ± 0.66
Hexadecane	226.44	14.39	1601		16.01	0.16 ± 0.02
Heptadecane	240.48	15.53	1701		17.01	5.69 ± 0.88
Nonadecane	268.52	17.64	1901		19.01	1.21 ± 0.20
Eicosane	282.55	18.62	2001		20.01	0.45 ± 0.07
Heneicosane	296.57	19.56	2101		21.01	11.08 ± 1.62
Docosane	310.61	20.46	2201		22.01	3.20 ± 0.45
Tricosene isomer	322.61	21.15	2280		22.80	0.32 ± 0.06
Tricosane	324.63	21.32	2297		22.97	19.84 ± 2.70
Tetracosane	338.65	22.14	2401		24.01	0.13 ± 0.02
Pentacosene isomer	350.66	22.74	2475		24.75	0.32 ± 0.06
Pentacosane	352.68	22.95	2501		25.01	0.55 ± 0.07
1-Heptacosene	378.72	24.24	2672	2688 [34]	26.72	0.31 ± 0.06
Heptacosane	380.73	24.46	2701		27.01	0.39 ± 0.05
1-Nonacosene	406.77	25.92	2870	2888 [34]	28.70	0.27 ± 0.06
Nonacosane	408.79	26.20	2901		29.01	1.17 ± 0.20
11-; 13-; 15-Methylnonacosane	422.81	26.47	2930	2932 [14]	29.30	0.12 ± 0.02
Triacontane	422.81	27.23	3002		30.02	0.06 ± 0.01
Hentriacontane	436.84	28.38	3104		31.04	1.24 ± 0.21
11-; 13-; 15-Methylhentriacontane	450.87	28.67	3127	3135 [14]	31.27	0.49 ± 0.11
12,16-Dimethyldotriacontane	478.92	30.28	3251	3157 [14]	32.51	0.37 ± 0.12
13-; 15-; 16-Methyltritriacontane	478.92	31.36	3326	3335 [14]	33.26	0.49 ± 0.15
13,17-Dimehtyltritriacontane	492.95	31.72	3350	3364 [14]	33.50	0.45 ± 0.07

MW: Molecular weight; RT (min): Retention time in minutes; RI: Retention index; Lit. RI [Ref]: Literature retention Index; SE: Standard error; ECL: Equivalent chain length (ECL = RI/100).

**Table 2:** Compounds isolated from Dufour’s gland of workers of *Oecophylla smaragdina.*

Compounds	MW	RT (min)	RI	Lit. RI [Ref]	ECL	Content (%)
						(Mean ± SE)
Decane	142.28	6.15	1104		11.04	1.02 ± 0.17
1-Undecene	154.29	7.53	1093	1090 [30]	10.93	tr
Undecane	156.31	7.67	1101		11.01	27.45 ± 3.98
Dodecane	170.33	9.17	1201		12.01	1.11 ± 0.19
1-Tridecene	182.35	10.39	1288	1288 [31]	12.88	tr
Tridecane	184.36	10.58	1301		13.01	7.21 ± 1.71
Pentadecane	212.41	13.19	1501		15.01	5.54 ± 1.26
Heptadecane	240.48	15.53	1701		17.01	8.72 ± 1.52
nonadecane	268.52	17.64	1901		19.01	2.15 ± 0.35
1-Heptadecanol	256.47	18.03	1941	1941 [35]	19.41	0.44 ± 0.06
Heneicosane	296.57	19.56	2101		21.01	16.07 ± 3.07
Docosane	310.61	20.46	2201		22.01	4.45 ± 0.86
Tricosane	324.63	21.32	2297		22.97	25.83 ± 4.01

MW: Molecular weight; RT (min): Retention time in minutes; RI: Retention index; Lit. RI [Ref]: Literature retention Index; SE: Standard error; ECL: Equivalent chain length (ECL = RI/100).

**Table 3:** Compounds isolated from poison gland of workers of *Oecophylla smaragdina.*

Compounds	MW	RT (min)	RI	Lit. RI [Ref]	ECL	Content (%)
						(Mean ± SE)
Decane	142.28	6.15	1104		11.04	0.48 ± 0.11
1-Undecene	154.29	7.53	1093	1090 [30]	10.93	tr
Undecane	156.31	7.67	1101		11.01	52.68 ± 12.10
Dodecane	170.33	9.17	1201		12.01	0.52 ± 0.12
1-Tridecene	182.35	10.39	1288	1288 [31]	12.88	0.31 ± 0.01
Tridecane	184.36	10.58	1301		13.01	4.38 ± 0.97
Pentadecene isomer	NA	13.01	1499		14.99	tr
Pentadecane	212.41	13.19	1501		15.01	3.18 ± 0.68
Heptadecane	240.48	15.53	1701		17.01	5.06 ± 1.05
nonadecane	268.52	17.64	1901		19.01	1.25 ± 0.25
1-Heptadecanol	256.47	18.03	1941	1288 [31]	19.41	4.10 ± 0.99
Heneicosane	296.57	19.56	2101		21.01	5.89 ± 1.79
Docosane	310.61	20.46	2201		22.01	1.70 ± 0.52
Tricosane	324.63	21.32	2297		22.97	15.65 ± 3.02

MW: Molecular weight; RT (min): Retention time in minutes; RI: Retention index; Lit. RI [Ref]: Literature retention Index; SE: Standard error; ECL: Equivalent chain length (ECL = RI/100).

**Table 4:** Compounds isolated from the head of the workers of *Oecophylla smaragdina.*

Compounds	MW	RT (min)	RI	Lit. RI [Ref]	ECL	Content (%)
						(Mean ± SE)
1-Hexanol	102.17	4.19	863	867 [44]	8.63	1.84 ± 0.29
Hexanoic acid	116.16	5.75	977	974 [45]	9.77	3.81 ± 0.59
4,6-Dimethyldecane	170.33	6.99	1057		10.57	0.10 ± 0.04
1-Octanol	130.23	7.22	1073	1068 [31]	10.73	0.46 ± 0.09
1-Undecene	154.29	7.53	1093	1090 [32]	10.93	0.01 ± 0.01
Undecane	156.31	7.67	1101		11.01	0.06 ± 0.02
2, 4, 9-trimethyldodecane	212.41	10.28	1280		12.80	0.09 ± 0.03
Heptadecane	240.48	15.53	1701		17.01	0.40 ± 0.07
Palmitic acid	256.42	18.24	1962	1964 [31]	19.62	5.04 ± 0.75
Linoleic acid	280.45	19.94	2143	2140 [46]	21.43	1.98 ± 0.36
Oleic acid	282.46	19.96	2146	2147 [47]	21.46	14.25 ± 2.92
Stearic acid	284.48	20.13	2164	2168 [48]	21.64	0.37 ± 0.22
11-; 13-Methylheptacosane	394.76	24.69	2728	2731 [49]	27.28	3.36 ± 0.55
Nonacosane	408.79	26.20	2901		29.01	2.41 ± 0.41
11-; 13-; 15-Methylnonacosane	422.81	26.47	2930	2932 [14]	29.30	11.07 ± 1.88
11,19-; 13,15-Dimethylnonacosane	436.84	26.73	2959		29.59	4.25 ± 0.70
12-; 13-; 14-; 15-Methyltriacontane	436.84	27.50	3026		30.26	2.98 ± 0.50
9,13-; 12,15-Dimethyltriacontane	450.87	27.79	3052		30.52	1.59 ± 0.26
Hentriacontane	436.84	28.38	3104		31.04	2.00 ± 0.35
11-; 13-; 15-Methylhentriacontane	450.87	28.67	3127	3135 [14]	31.27	31.59 ± 5.75
11,15-; 13,14-Dimethylhentriacontane	464.89	28.99	3153	3163 [14]	31.52	20.89 ± 3.84
12-; 14-; 15-; 16-Methyldotriacontane	464.89	29.94	3225	3231 [14]	32.25	3.65 ± 0.63
12,16-Dimethyldotriacontane	478.92	30.28	3251	3257 [14]	32.51	3.37 ± 0.59
13-; 15-; 16-Methyltritriacontane	478.92	31.36	3326	3335 [14]	33.26	21.64 ± 4.11
14,17-Dimehtyltritriacontane	492.95	31.72	3351	3364 [14]	33.50	24.79 ± 4.85
14,18-Dimethyltetratriacontane	506.97	33.26	3449	3456 [14]	34.49	1.54 ± 0.29
13-; 15-; 17-Methylpentatriacontane	506.97	34.53	3524	3534 [14]	35.24	0.92 ± 0.50
13,17-; 15,19-Dimethylpentatriacontane	506.97	34.95	3547	3559 [14]	35.47	2.18 ± 0.90

MW: Molecular weight; RT (min): Retention time in minutes; RI: Retention index; Lit. RI [Ref]: Literature retention Index; SE: Standard error; ECL: Equivalent chain length (ECL = RI/100).

**Table 5:** Headspace volatile compounds emitted by workers of *Oecophylla smaragdina.*

Compounds	MW	RT (min)	RI	Lit. RI [Ref]	ECL	Content (%)
						(Mean ± SE)
1-Hexanol	102.17	4.19	863	867 [45]	8.63	0.93 ± 0.29
Decane	142.28	6.15	1104		11.04	0.62 ± 0.09
*p*-Cymene	134.22	6.57	1031	1030 [53]	10.31	1.81 ± 0.73
Limonene	136.23	6.63	1035	1044 [30]	10.35	7.11 ± 2.05
γ-Terpinene	136.23	7.10	1065	1062 [54]	10.65	1.34 ± 0.46
1-Octanol	130.23	7.22	1073	1068 [31]	10.73	1.17 ± 0.51
Dihydromyrcenol	156.27	7.25	1074	1076 [55]	10.74	35.34 ± 10.61
Undecane	156.31	7.67	1101		11.01	31.17 ± 7.53
Nonanal	142.24	7.76	1107	1108 [56]	11.07	0.67 ± 0.13
Dodecane	170.33	9.17	1201		12.01	1.05 ± 0.19
Tridecane	184.36	10.58	1301		13.01	3.77 ± 0.77
Tetradecane isomer	196.37	11.70	1395		13.95	0.31 ± 0.04
Tetradecane	198.39	11.92	1401		14.01	1.08 ± 0.13
Pentadecane	212.41	13.19	1501		15.01	5.37 ± 0.96
Hexadecane	226.44	14.39	1601		16.01	1.76 ± 0.42
Heptadecane	240.48	15.53	1701		17.01	6.49 ± 1.07

MW: Molecular weight; RT (min): Retention time in minutes; RI: Retention index; Lit RI [Ref]: Literature retention Index; SE: Standard error; ECL: Equivalent chain length (ECL = RI/100).

**Table 6:** Trail compounds of the workers of *Oecophylla smaragdina*.

Compounds	MW	RT (min)	RI	Lit. RI [Ref]	ECL	Content (%)
						(Mean ± SE)
Limonene	136.23	6.63	1035	1044 [30]]	10.35	0.80 ± 0.11
Undecane	156.31	7.67	1101		11.01	28.30 ± 4.33
Tridecane	184.36	10.58	1301		13.01	1.90 ± 0.32
Tetradecane	198.39	11.92	1401		14.01	6.36 ± 1.11
Heptadecane	240.48	15.53	1701		17.01	1.88 ± 0.29
Heneicosane	296.57	19.56	2101		21.01	5.45 ± 0.73
Docosane	310.61	20.46	2201		22.01	1.69 ± 0.24
Tricosane	324.63	21.32	2297		22.97	9.24 ± 0.99
heptacosane	380.73	24.46	2701		27.01	3.24 ± 0.66
Nonacosane	408.79	26.20	2901		29.01	15.35 ± 1.99
Hentriacontane	436.84	28.38	3104		31.04	25.79 ± 4.02

MW: Molecular weight; RT (min): Retention time in minutes; RI: Retention index; Lit. RI [Ref]: Literature retention Index; SE: Standard error; ECL: Equivalent chain length (ECL = RI/100).
